# Associations between political orientation and allyship: Evidence from potential allies and their LGBTQ+ close others

**DOI:** 10.1038/s41598-026-42213-8

**Published:** 2026-03-01

**Authors:** Haley Bock, Jacqueline M. Chen, Samantha Joel

**Affiliations:** 1 Department of Psychology, University of Utah, Salt Lake City, UT USA; 2 Western University, London, ON Canada

**Keywords:** Human behaviour, Risk factors

## Abstract

**To support the LGBTQ+ community**,** many straight**,** cisgender individuals position themselves as** ***allies*** **to their cause. It is possible that those identifying as liberal may champion LGBTQ+ causes more passionately than those identifying as conservative**,** though it is also possible that liberals’ self-perceptions do not align with how LGBTQ+ individuals perceive them. In this study**,** we systematically investigated the relationship between political orientation and allyship to the LGBTQ+ community. We recruited 378 dyads composed of a cisgender**,** straight individual and an LGBTQ+ close other. Findings suggested that self-perceptions of allyship (from cisgender**,** straight individuals) were largely consistent with evaluations from LGBTQ+ close others. In line with our expectations**,** on average**,** liberals (compared to conservatives) both viewed themselves and were perceived as better allies. However**,** there was a small but significant tendency for liberals to** ***overestimate*** **their allyship relative to conservatives. In addition**,** exploratory analyses revealed other-perceived allyship was positively associated with higher interpersonal trust**,** underscoring allyship’s importance in close relationships. These findings contribute to a growing understanding of the ideological and interpersonal antecedents of allyship and inform strategies for fostering stronger**,** more authentic relationships with the LGBTQ+ community.**

The stage 1 protocol for this Registered Report was accepted in principle on 11/15/2024. The protocol, as accepted by the journal, can be found at: 10.17605/OSF.IO/2Q7W6.

**Supplementary Information:**

The online version contains supplementary material available at 10.1038/s41598-026-42213-8.

## Introduction

Nearly 8% of the United States population identifies as LGBTQ+ (lesbian, gay, bisexual, transgender, queer, and any other gender identity or sexual orientation not included in the acronym)^1^. Amidst ongoing public debates, such as those surrounding restrictions on transgender athletes’ participation in sports^2^, members of this community encounter unique challenges. Compounded by experiences of discrimination, such challenges can contribute to higher rates of mental health disorders^3^ and suicide^4^ among LGBTQ+ individuals compared to their straight, cisgender counterparts. Ultimately, addressing these challenges and disparities necessitates a thorough examination of the underlying factors driving others’ support for the LGBTQ+ community. As interest in support for the LGBTQ+ community grows, the idea of allyship has gained traction in public discourse and academic research. *Allyship* is broadly defined as actions of individuals from relatively more advantaged social backgrounds that support the interests of individuals from relatively disadvantaged social backgrounds^5^. Allyship to LGBTQ+ people by people outside of the community is multifaceted and includes being nonprejudiced towards LGBTQ+ people, taking action against LGBTQ+ discrimination, and having humility for one’s perspective during discussions of LGBTQ+ issues^6^. In line with other work on allyship^6–9^, we believe allyship is best understood from the perspective of the social group it aims to support, rather than as a self-proclaimed identity. If allyship is indeed successful, then members of the group being offered allyship should perceive it as *true* allyship. Indeed, among LGBTQ+ individuals, the true allyship of close others is associated with positive outcomes, such as improved psychological well-being^6^, felt authenticity^10^, and a stronger sense of belonging^11^. It is therefore important to document the underlying processes that promote or diminish individuals’ true allyship to the LGBTQ+ community.

In the U.S., political debates on LGBTQ+ issues suggest that there are strongly polarized attitudes toward the community^12^. However, research has not directly examined whether liberal or conservative individuals are better allies to the LGBTQ+ community. Past research shows that politically liberal individuals tend to *report* being more supportive of marginalized groups in general compared to conservatives^13,14^. Yet, self-designated allyship may not necessarily be recognized as such by members of the marginalized group. It is an open question whether LGBTQ+ individuals themselves perceive liberals versus conservatives as being better or worse allies. Further, it is yet unknown how well peoples’ perceptions of their own allyship align with, or diverge from, their true allyship as perceived by their LGBTQ+ family, friends, or coworkers. We argue this approach provides the opportunity to examine *biased* evaluations (i.e., over- or under-estimations of self-perceived allyship relative to true allyship), which may have distinct consequences for outcomes for marginalized group members^15^. The current research systematically investigated the association between political orientation and LGBTQ+ allyship, as perceived by the straight, cisgender allies and by the LGBTQ+ individuals in their social circles. We evaluated the extent to which self-perceptions of allyship align with other-perceptions (i.e., true allyship) and the degree of discrepancy between them (i.e., bias).

Understanding the role of political orientation in self-perceived and other-perceived allyship contributes to the diversity science, political psychology, and close relationships literatures. With respect to diversity science, understanding the individual differences that predict allyship, and potential moderating factors, is important for understanding social justice behaviors, and cross-group relationships. Also, understanding the relationship between political orientation and allyship could help guide educational trainings by identifying personalized areas for improvement. For instance, pinpointing how allyship qualities (e.g., humility) vary between (or within) political orientation can lead to more effective, tailored training on allyship. Relevant to political psychology, this research improves our understanding of whether, and potentially why, liberals are better allies to the LGBTQ+ community compared to conservatives. Finally, this research expands the literature on close relationships by improving our understanding of cross-group relationships, which are essential for reducing prejudice^16^. Identifying how self-perceptions versus other-perceptions of allyship align or diverge across these group boundaries provides further insights into these types of relationships.

A substantial body of research supports the notion that liberal individuals perceive themselves as better allies to the LGBTQ+ community than conservative individuals. One key finding is that liberals tend to have stronger motivations for egalitarianism than conservatives^17,18^, potentially driving their propensity for allyship^13^. Further, both political parties perceive the LGBTQ+ community as ideologically liberal^19^, and this perception may simultaneously lead liberal individuals to defend the community and conservative individuals to exhibit intolerance towards it. Liberals also report participating in more collective action for marginalized groups than conservatives^20^. For example, in the workplace, liberal employees report engaging in more self-reflection on privilege, relational support for underrepresented colleagues, and organizational efforts to change structures compared to conservative employees^21^. In addition, research^22^ has demonstrated that conservatives (vs. liberals) endorse more essentialist beliefs about LGBTQ+ people. This tendency may contribute to their support for anti-LGBTQ+ legislation^23^, such as restroom policies based on assigned sex at birth^24^. Collectively, these findings suggest that liberals, viewing the LGBTQ+ community as tied to their political identity, may strive for more positive intergroup relations and are more likely than conservatives, who are less egalitarian and more essentialist, to be supportive of the LGBTQ+ community as allies.

Other characteristics of political parties may offer additional insight into their relationships with the LGBTQ+ community. Religiosity, which is strongly associated with political conservatism^25^, plays an important role in how cisgender, straight people interact with LGBTQ+ people. Historically, highly religious people have harbored negative attitudes toward the LGBTQ+ community, such as believing same-sex attraction is a perversion^26^. In the U.S, opposition to LGBTQ+ rights and their societal standing is particularly prevalent within Christian communities^27,28^. Conversely, liberals tend to have more interactions with LGBTQ+ people compared to conservatives^29^, which can strengthen ally identity^30^ and intentions for collective action^31,32^. Indeed, lower levels of religiosity and increased exposure to members of the LGBTQ+ community may prepare liberal individuals to be better allies than conservative individuals.

It is important to note that the evidence discussed thus far focuses on self-reported surveys, revealing that liberals report stronger egalitarian motivations^17,18^, fewer prejudiced attitudes^22,33^, greater interest in collective action^14,20^, and more contact with LGBTQ+ people compared to conservatives^29^. Nonetheless, it remains unclear whether these self-reported tendencies actually map onto true allyship from the perspective of LGBTQ+ individuals themselves.

Social psychology has long documented the influence of self-enhancement and protection biases on behavior (see for review^34^). Such tendencies for people to exaggerate their virtues and downplay their weaknesses may pose different consequences for how liberals and conservatives view their beliefs about and actions (or inactions) towards the LGBTQ+ community. Liberals, who tend to hold a more favorable view of allyship than conservatives^35^, may be more driven to see themselves as good allies to LGBTQ+ individuals. While both liberals and conservatives may *overestimate* their allyship to the LGBTQ+ community due to universal tendencies for a positive self-view, liberals may be especially prone due to such ideological motivations.

This possible gap between the self-perception and observers’ perception of allyship becomes clearer when considering existing literature on social action, where positive intentions do not always translate into positive intergroup behaviors^36–38^. Individuals who strive to be good allies may make mistakes (e.g., failing to acknowledge privilege) in their efforts, which can result in them being perceived as ineffective^7^, less appreciated for their actions^39^, and viewed as contributors to burnout for the marginalized group they purport to help^40^. Such tendencies may reflect *performative allyship* (see for review^41^), where individuals can engage in allyship to enhance their own self-image, rather than from a genuine desire to improve the status of marginalized groups^14^. In these cases, biased self-perceptions of allyship may emerge, with LGBTQ+ individuals recognizing these self-oriented (vs. other-oriented) motivations^7^. Taken together, these findings highlight the possibility that well-intended cisgender, straight liberals might not actually *behave* as the allies they imagine themselves to be. Measuring true allyship through others’ perceptions of an individual’s allyship provides a more comprehensive understanding of how strongly political orientation is associated with allyship.

To our knowledge, only one previously published study has taken a dyadic approach to the study of LGBTQ+ allyship^6^. In a sample of young adult LGBTQ+ individuals and their straight, cisgender roommates, a strong correlation between allyship ratings was observed across dyads (Study 4). This finding tentatively suggests that individuals’ assessments of their own allyship were consistent with LGBTQ+ individuals’ perceptions. However, as participants were pairs of cohabitants, the associations documented in this research may overestimate the relationship between self-perceived and other-perceived allyship across different types of social relationships between LGBTQ+ individuals and cisgender, straight others. Further, raw correlations can mask more complex associations, particularly when examining accuracy in dyadic contexts^42^ and other possible moderating factors (e.g., political orientation). A thorough answer to the research question requires a broader range of relationship types beyond cohabitants, as well as the inclusion of additional variables that may influence perceptions of allyship.

The current study examined the extent to which individuals’ political orientation is associated with both self-perceived and other-perceived allyship to the LGBTQ+ community among people living in the U.S. Our methods and analytic plan were all pre-registered. We recruited participants who nominated a person with whom they have regular contact, creating dyads. Among recruited participants, LGBTQ+ identifying participants were asked to nominate a cisgender, straight person, while cisgender, straight identifying participants were asked to nominate a person who identified as LGBTQ+. LGBTQ+ participants rated the cisgender, straight person on their level of allyship (i.e., other-perceived allyship), while cisgender, straight participants rated themselves on their level of allyship (i.e., self-perceived allyship) and their political orientation. The following Research Questions (RQ) and their corresponding Hypotheses (H) were tested, as well as several Exploratory Analyses (EA):

**RQ1: How is political orientation associated with allyship to the LGBTQ+ community?** We predicted that political orientation would predict self-perceived allyship, such that liberal individuals would rate themselves as better allies than conservative individuals (H1a). We further predicted that political orientation would predict other-perceived allyship, such that LGBTQ+ people would rate liberal individuals better on allyship than conservative individuals (H1b).

**RQ2: To what extent are there biases in perceptions of LGBTQ+ allyship among liberals and conservatives?** We expected a general positivity bias whereby cisgender, straight individuals overestimate the extent to which they are good allies, as rated by LGBTQ+ individuals (H2a). Further, we expected this positivity bias to be moderated by political orientation, such that liberal (vs. conservative) individuals would be particularly likely to overestimate their own allyship (H2b).

**Exploratory Analyses.** We pre-registered several exploratory analyses. These analyses were considered exploratory because they were not accompanied by specific, directional hypotheses at the time of pre-registration. Nonetheless, we believed they addressed important questions worthy of investigation. For detailed descriptions and justifications of all exploratory analyses, please refer to our OSF page (https://osf.io/2q7w6/overview?view_only=b9f680082390416a90c3e456aeee29b9).

We suspected there could be additional factors that weigh into the relationship between political orientation and allyship to LGBTQ+ individuals. First, conservative attitudes about LGBTQ+ issues may be becoming more positive, particularly among younger generations^43^, and LGBTQ+ identification is also more common in younger (vs. older) generations^1^. Accordingly, it may be that younger individuals, regardless of political orientation, may have a more inclusive stance towards the LGBTQ+ community than older individuals (EA1).

The degree of closeness between LGBTQ+ individuals and their potential allies could also have implications for perceptions of allyship. Positive illusions, which involve accentuating positive traits and downplaying negative ones, are common in maintaining both romantic^44,45^ and platonic relationships^46^. Therefore, when LGBTQ+ individuals have close relationships with straight, cisgender individuals, they may exaggerate their strengths and overlook their flaws regarding allyship. Consistent with this idea, research^6^ has shown that LGBTQ+ individuals’ feeling close to their roommate one week predicted perceiving them to be a better ally the following week, even after accounting for perceived allyship the week before. Regardless of political orientation, LGBTQ+ individuals who perceive a close connection with a cisgender, straight individual may thus rate their allyship more favorably than those with whom they have a distant relationship (EA3).

Finally, one way to further our understanding of biased self-perceptions is by examining their association with relevant outcomes for our participants’ relationships. To do this, we measured LGBTQ+ participants’ perceptions of interpersonal trust^47^ with the cisgender, straight person in their social circle. We wondered whether a positivity bias (overestimating allyship) would predict reduced trust, supporting the idea that this bias reflects ineffective or performative allyship. Negativity biases (underestimating allyship) may be less intuitive. On one hand, a negativity bias could predict increased trust, reflecting humility—a core aspect of true allyship^6^. On the other hand, research suggests that aligned perceptions may have the strongest impact on recipients of allyship^15^. In that case, trust would remain stable with matched perceptions or decrease in response to underestimations. Exploring the impact of bias on trust was the focus of our seventh exploratory analysis (EA7).

## Method

### Ethics information

The Institutional Review Board at the University of Utah approved this study. Data collection and data security procedures were performed in accordance with IRB guidelines and regulations. Informed consent was obtained from all participants. On average, Phase 1 and Phase 2 surveys each took 15 min to complete, and all participants were paid $3.00 each, which is roughly equivalent to $12/hour.

## Design

Refer to Table 1 for descriptions of each research question, hypothesis, and associated analysis strategy.

**Table 1:** Design Table.

Question	Hypothesis (if applicable)	Sampling Plan (e.g., power analyses)	Analysis Strategy	Interpretation given to different outcomes
*RQ1: How is political orientation associated with allyship for the LGBTQ+ community?*	H1a_0_: Liberals will not rate themselves as better allies relative to conservatives. H1a_1_: Liberals will rate themselves as better allies relative to conservatives. H1b_0_: Liberals will not be perceived as better allies than conservatives. H1b_1_: Liberals will be perceived as better allies than conservatives.	α = 5%, minimum power = 95%, two-sided; able to detect *r* = .18 with *N* = 756 (378 dyads)	Linear regression: 1. Self-perceived allyship regressed on political orientation. 2. Other-perceived allyship regressed on political orientation. Regressions will be conducted with and without controlling for religiosity and frequency of contact with nominee. Separate regressions will be conducted for different measures of political orientation.	If the relationship between political orientation and self-perceived allyship is statistically significant (*p* < .05), we will conclude finding support for H1a. If the relationship between political orientation and other-perceived allyship is statistically significant (*p* < .05), we will conclude finding support for H1b.
RQ2*: How is political orientation associated with bias in the perceptions of allyship to the LGBTQ+ community?	H2a_0_: There will be no bias between self-perceptions and other perceptions of allyship H2a_1_: There will be a general positivity bias whereby cisgender, straight individuals overestimate the extent to which they are good allies, as rated by LGBTQ+ individuals H2b_0_: Liberals will be no more likely to overestimate their allyship relative to conservatives H2b_1_: Liberal (vs. conservative) individuals will overestimate their own allyship, after accounting for other-perceived allyship.	α = 5%, minimum power = 95%; able to detect *d* = 0.19, *f*^2^ = 0.05 with *N* = 756 (378 dyads)	One Sample *t*-test: 1. Evaluate whether difference between self-perceived allyship and other-perceived allyship is statistically significantly different from 0. Linear regression: 1. Bias (difference between self-perceived and other-perceived allyship) will be regressed on political orientation. 2. Self-perceived allyship will be regressed on other-perceived allyship, political orientation, and their interaction. Regression (model 1) will be conducted with and without controlling for religiosity and frequency of contact with nominee. Separate analyses will be conducted for different measures of political orientation.	If the *t* is positive and statistically significant (*p* < .05), we will conclude finding support for H2a. If the main effect of political orientation is statistically significant in either regression model (*p* < .05) and in the positive direction (after accounting for the effect of other-perceived allyship in second model), we will conclude finding support for H2b.

Note. *These analyses deviate from our pre-registered plan. Specifically, we had to depart from our original analysis strategy for RQ2 due to its statistical infeasibility. A summary of these deviations is provided in the “Analysis Strategy” section, and a detailed account, including each specific change, our rationale, and potential implications for our findings, is available on our OSF page (https://osf.io/2q7w6/?view_only=b9f680082390416a90c3e456aeee29b9).

## Sampling plan

This section includes information on the sample characteristics, exclusion criteria, and power analysis.

## Participants

Participants were recruited from CloudResearch Connect (*n* = 955) and a university in the Mountain West US (*n* = 162), with a total of 1,117 Phase 1 participants. We received responses from approximately 46.28% of individuals nominated by Phase 1 participants (*n*_Connect_ = 433, *n*_University_ = 84, total Phase 2 *n* = 517). Those who did not form a complete dyad (*n* = 610) were excluded from analyses. Following our pre-registration, we excluded participants who did not pass both attention checks or had missing data for at least one attention check (*n*_Fail_ = 95, *n*_Missing_ = 105).

Participants who identified as something other than heterosexual who were nominated by LGBTQ+ identifying participants (*n* = 7) were ineligible for inclusion as well. Using the following criteria, we also flagged dyads as possible self-nominators and excluded them from our final sample (*n* = 28): (1) same IP addresses, (2) same GPS coordinates, (3) start of Phase 2 survey within a minute of end of Phase 1 survey, and (4) report they are not currently living together.

We had a final sample of 378 cisgender, straight/LGBTQ+ dyads. Using the “pwr” package^48^ in R studio, we determined that this sample is powered to detect relatively small to medium effects (i.e., *r* = .18, *f*^2^ = 0.05, *d* = 0.19) at 95% power. Data were collected from January to March of 2025.

The sample was predominantly White (70.1%), with a majority identifying as women (45.0%–58.7%). Participants had an average age of 30.56-33.50 (*SD*s = 9.95-12.20). Most participants were college-educated (32.3%–37.6%), identified as lower-middle class (24.3%–27.0%), were employed full-time (52.9%–59.8%), and resided in the Western United States (33.9%–35.2%). Among cisgender, straight participants, most participants identified as Democrats (69.0% Democrats, 30.7% Republicans). Roughly similar rates of self-identified Democrats and Republicans were excluded based on our exclusion criteria (see our supplemental file for additional information). Table 2 includes demographic characteristics of our final sample.

**Table 2:** *Demographics of sample by LGBTQ+ identity*.

Category	% (*n*)	
	LGBTQ+	Cishet
Race		
White	70.1 (265)	70.1 (265)
Asian	7.4 (28)	10.3 (39)
Black	8.2 (31)	7.7 (29)
Latin	7.9 (30)	7.7 (29)
Biracial/Mixed	5.0 (19)	3.4 (13)
Middle Eastern	1.1 (4)	0.5 (2)
Native American	0.3 (1)	
Other racial identity		0.3 (1)
Born in the US		
Yes	95.2 (360)	94.7 (358)
No	4.8 (18)	5.3 (20)
Gender		
Woman	58.7 (222)	45.0 (170)
Man	31.0 (117)	55.0 (208)
Non-binary	9.0 (34)	
I don’t know	0.5 (2)	
Other gender	0.8 (3)	
Sex		
Female	65.1 (246)	45.0 (170)
Male	34.9 (132)	55.0 (208)
Sexual Orientation		
Heterosexual	3.2 (12)	100.0 (378)
Bisexual	53.4 (202)	
Gay	35.2 (133)	
Other sexual orientation	8.2 (31)	
Political Party		
Democrat		69.0 (261)
Republican		30.7 (116)
Missing		0.3 (1)
Education		
Bachelor’s degree	32.3 (122)	37.6 (142)
Some college	25.1 (95)	23.8 (90)
High school graduate - diploma or equivalent	15.6 (59)	15.1 (57)
Associate degree	12.7 (48)	9.8 (37)
Master’s degree	10.3 (39)	8.7 (33)
Professional degree	2.1 (8)	1.9 (7)
Doctoral degree	0.5 (2)	1.9 (7)
Less than a high school diploma	1.3 (5)	1.1 (4)
Prefer not to say		0.3 (1)
Occupation		
STEM	19.3 (73)	30.9 (117)
Healthcare & Medicine	11.1 (42)	9.8 (37)
Arts & Humanities	11.9 (45)	4.3 (16)
Retail & Hospitality	10.1 (38)	7.5 (28)
Business & Finance	9.5 (36)	15.1 (57)
Skilled Trades	8.0 (30)	9.0 (34)
Education	6.9 (26)	6.3 (24)
Government & Law Enforcement	2.7 (10)	5.6 (21)
Other/Unspecified	15.9 (60)	11.6 (44)
Income		
Lower-Middle ($30k–$59k)	24.3 (92)	27.0 (102)
Middle ($60k–$99k)	24.3 (92)	25.1 (95)
Low Income (<$30k)	20.9 (79)	14.8 (56)
Upper-Middle ($100k–$149k)	13.8 (52)	15.3 (58)
High ($150k or more)	13.7 (52)	16.4 (62)
Prefer not to say	2.9 (11)	1.4 (5)
Employment Status		
Full-time	52.9 (200)	59.8 (226)
Part-time	15.3 (58)	14.0 (53)
Student	15.3 (58)	12.4 (47)
Unemployed	5.3 (20)	4.5 (17)
Not in paid work (e.g., homemaker, disabled)	4.5 (17)	3.2 (12)
Business Owner	4.8 (18)	2.9 (11)
Retired	1.3 (5)	2.6 (10)
Prefer not to say	0.5 (2)	0.5 (2)
Region in the US		
West	33.9 (128)	35.2 (133)
South	30.7 (116)	31.0 (117)
Northeast	19.3 (73)	18.0 (68)
Midwest	15.9 (60)	15.1 (57)
Puerto Rico	0.3 (1)	0.8 (3)

Note. Cishet refers to cisgender, heterosexual (straight) respondents. Percentages are calculated from the total number of participants in each identity category (*N* = 378).

Among LGBTQ+ identifying participants, participants identified with a range of sexual and gender identities. The majority identified as bisexual (*n* = 192) alone, or in combination with other labels (e.g., questioning, queer, pansexual), followed by those who identified as gay or lesbian (*n* = 75) alone, or in combination with other labels, queer only (*n* = 5), pansexual only (*n* = 5), asexual only (*n* = 7), or transgender only (*n* = 8). Remaining participants selected complex or less frequent combinations that did not fit into one of these categories.

LGBTQ+ participants also completed measures about their relationship with their cisgender, straight close other. There were a range of relationships observed in the data, but most dyads were friends (*n* = 210), cohabiting (*n* = 212), and had known each other for an average of 11.50 years (*SD* = 9.81, *range* = 0–46 years). Our supplemental file includes more information regarding the specific relationships among the dyads in our data.

## Materials

All materials, including our codebook, are included on our OSF page (https://osf.io/2q7w6/overview?view_only=b9f680082390416a90c3e456aeee29b9).

### Political orientation

Political orientation of the cisgender, straight individual was measured with two items, “In terms of [economic/social] issues, how would you describe your political attitudes and beliefs?” from 1 (*Very conservative*) to 7 (*Very liberal*). The average of the two items (*M* = 4.64, *SD* = 1.74, *range* = 1.00–7.00, α = 0.91) was used for analyses.

## Allyship

Allyship for the LGBTQ+ community was measured using an 11-item^6^ scale. For LGBTQ+ identifying participants, items referred to the cisgender, straight close other (e.g., “They speak out against LGBTQ+ discrimination”), whereas for cisgender, straight identifying participants, items referred to themselves (e.g., “I vocally support the LGBTQ+ community”). All items were rated from 1 (*Not true*) to 7 (*Extremely true*). After all items were averaged, self-perceived allyship (*M* = 5.27, *SD* = 1.07, *range* = 2.09–7.00, α = 0.90) and other-perceived allyship composites (*M* = 5.30, *SD* = 1.09, *range* = 2.00–7.00, α = 0.91) were created.

## Religiosity

Religiosity was measured using a 5-item scale (α = 0.90)^28^. Items include a global assessment of how religious a person is (i.e., “How religious are you?” from 1 (*Not at all religious*) to 7 (*Extremely religious*)) as well as items referring to how important religion is to a person’s self-image (i.e., “My religion is an important reflection of who I am”). Excluding the global assessment item, the remaining items were rated from 1 (*Strongly disagree*) to 7 (*Strongly agree*). After all items were averaged, cisgender, straight respondents’ ratings (*M* = 3.58, *SD* = 1.90, *range* = 1.00–7.00, α = 0.90) and LGBTQ+ identifying respondents’ ratings (*M* = 3.00, *SD* = 1.67, *range* = 1.00–7.00, α = 0.91) were created. Cisgender, straight ratings of religiosity were used as a covariate in the planned analyses.

### Frequency of contact

Frequency of contact was measured with one item, “How often do you have contact with [nominee/the person who nominated you]?”. Options ranged from 1 (*A few times a year or less*) to 6 (*Every day*). This item was only administered in the LGBTQ+ identifying persons’ survey and reflected relatively frequent contact (*M* = 4.95, *SD* = 1.12, *range* = 2.00–6.00). This measure was included as a covariate in the planned analyses.

### Procedure

The study was conducted in two phases where participants recruited in the Phase 1 would nominate a close other to complete the Phase 2 survey, creating dyads.

**Phase 1.** Participants first completed informed consent. They were then asked to nominate someone they have regular contact with (the “nominee”) to complete a similar survey. LGBTQ+ participants were instructed to nominate a cisgender, straight individual, while cisgender, straight participants were asked to nominate an LGBTQ+ individual. Survey measures were randomized, except those referring to the nominee, which appeared only after the nomination prompt. The timing of the nomination prompt (before or after individual difference measures) was counterbalanced.

***LGBTQ+ participants.*** Participants provided demographic information, as well as completed measures assessing their nominee’s allyship and characteristics of their relationship with the nominee (e.g., relationship type, frequency of contact, perceived closeness). They also responded to items assessing demographics about their nominee (including perceived political orientation). Additional survey items included a measure of interpersonal trust^47^ and filler items on social and political issues. Other measures not described here are included in full on our OSF page (https://osf.io/2q7w6/overview?view_only=b9f680082390416a90c3e456aeee29b9).

***Cisgender***,*** straight participants.*** Participants completed demographics and a measure of political orientation. They rated their own allyship using the same scale as LGBTQ+ participants and completed the same filler items.

**Phase 2.** Nominees completed an identical survey, excluding the nomination prompt. Items referencing the Phase 1 participant were framed as referring to “the person who nominated you.”

### Recruitment approach

To mitigate potential bias in nominations (e.g., LGBTQ+ participants nominating more liberal individuals), we split recruitment evenly between LGBTQ + and cisgender, straight participants. We anticipated LGBTQ+ individuals would more easily nominate cisgender, straight contacts than vice versa. To maximize diversity in cisgender, straight participants’ political orientation, we used CloudResearch Connect to screen cisgender, straight participants, targeting 30% Republican representation. Another 30% were not screened on political orientation. After reaching 60% of the desired Phase 1 cisgender, straight sample, we reviewed the political distribution and adjusted recruitment (e.g., screening for moderates) to correct imbalances. Recruitment from the local university’s subject pool did not include any screening.

### Analytic strategy

Table 1 outlines the specific statistical models used to address our focal research questions (RQ1 and RQ2). To examine RQ1, we tested whether political orientation predicted levels of allyship (both self-perceived and other-perceived) using linear regression models that controlled for religiosity and frequency of contact. For RQ2, we first conducted a one-sample *t*-test to assess whether, on average, cisgender, straight individuals’ self-perceptions of allyship differed from how they were perceived by LGBTQ+ close others. Then, we examined whether political orientation was a significant predictor of the difference between allyship ratings (i.e., bias). Finally, we tested whether political orientation moderated the association between other-perceived and self-perceived allyship using a linear regression model with an interaction term (political orientation × other-perceived allyship).

All analyses were conducted in R Studio^49^. Table 3 presents descriptive statistics and bivariate correlations among key continuous variables. Notably, cisgender, straight participants’ self-reported political orientation strongly aligned with LGBTQ+ participants’ perceptions of their political orientation, *r*(372) = 0.82, 95% CI [0.78, 0.85], *p* < .001.

**Table 3:** *Means*,* standard deviations*,* and correlations of key variables with confidence intervals*.

Variable	M	SD	1	2	3	4	5	6
1. SP Political Orientation	4.64	1.74						
2. OP Political Orientation	4.85	1.83	0.82***					
			[0.78, 0.85]					
3. SP Allyship	5.27	1.07	0.53***	0.44***				
			[0.45, 0.60]	[0.36, 0.52]				
4. OP Allyship	5.30	1.09	0.40***	0.43***	0.62***			
			[0.31, 0.48]	[0.34, 0.51]	[0.56, 0.68]			
5. Religiosity	3.58	1.90	− 0.36***	− 0.30***	− 0.17**	− 0.16**		
			[-0.45, − 0.27]	[-0.39, − 0.21]	[-0.26, − 0.07]	[-0.25, − 0.06]		
6. Frequency of Contact	4.95	1.12	0.16**	0.17***	0.14**	0.25***	− 0.15**	
			[0.06, 0.26]	[0.07, 0.27]	[0.04, 0.24]	[0.15, 0.34]	[-0.25, − 0.05]	
7. Bias	-0.03	0.94	0.14**	0.00	0.41***	− 0.46***	− 0.01	− 0.13*
			[0.04, 0.24]	[-0.10, 0.10]	[0.32, 0.49]	[-0.53, − 0.37]	[-0.11, 0.09]	[-0.23, − 0.03]

*Note. M* and *SD* are used to represent mean and standard deviation, respectively. Values in square brackets indicate the 95% confidence interval for each correlation. SP = Self-perceived, OP = Other-perceived. Religiosity reflects cisgender, straight reports and Frequency of Contact reflects LGBTQ+ identifying respondents’ reports. Bias is a difference score of SP and OP allyship scores, with higher scores reflecting over-estimation. ***p < .001, **p < .01, *p < .05.

The data were structured in wide format (one row per dyad). All regression models were evaluated for compliance with the assumptions of ordinary least-squares regression. As pre-registered, when assumptions were violated, we applied appropriate corrections (e.g., Tukey’s ladder of re-expression and the bulging rule; see review^50^) and reported the unadjusted results in-text and the adjusted results in a supplemental file for transparency. Outliers (± 3 SD from the mean) were winsorized to the next most extreme value.

We also ran supplemental analyses using a binary political orientation variable (Republican vs. Democrat). Because these results were consistent with the results using the continuous measure, we do not discuss these analyses further. Analyses for RQ1 and RQ2 included religiosity and frequency of contact with LGBTQ+ close others as covariates, given their theoretical relevance to both political orientation and allyship.

### Summary of deviations from pre-registration

We had originally planned to use an adapted version of the Truth and Bias Model^42^ to examine questions related to the alignment (vs. discrepancy) between allyship ratings and the potential influence of political orientation. However, these preregistered models produced evidence of statistical singularity—perfect linear dependence among the predictors—which made the produced statistical parameters unreliable. Further inspection revealed that our data lacked sufficient information (at least three unique sources of information measured using the same scale) to support the Truth and Bias Model, due to the asymmetry of the design (LGBTQ+ individuals rate allies, but allies do not rate LGBTQ+ individuals on allyship). We thus had to modify our analytic approach^51^. A detailed summary of these deviations is available on OSF (https://osf.io/2q7w6/overview?view_only=b9f680082390416a90c3e456aeee29b9).

## Results

Regression results for all focal hypotheses are included in Table 4.

**Table 4:** *Focal regression models and coefficients*.

Hypothesis	Model	Predictor	b	SE	95% CI	β	*p*
**H1a**	SP allyship ← Political orientation	(Intercept)	3.75	0.13	[3.49, 4.01]		< 0.001
		Political Orientation	0.33	0.03	[0.27, 0.38]	0.53	< 0.001
	With covariates	(Intercept)	3.38	0.28	[2.83, 3.93]		< 0.001
		Political Orientation	0.33	0.03	[0.27, 0.39]	0.54	< 0.001
		Religiosity	0.03	0.03	[-0.03, 0.08]	0.04	0.346
		Contact Frequency	0.05	0.04	[-0.03, 0.14]	0.06	0.218
**H1b**	OP allyship ← Political orientation	(Intercept)	4.14	0.15	[3.85, 4.43]		< 0.001
		Political Orientation	0.25	0.03	[0.19, 0.31]	0.40	< 0.001
	With covariates	(Intercept)	3.30	0.31	[2.69, 3.91]		< 0.001
		Political Orientation	0.23	0.03	[0.17, 0.29]	0.37	< 0.001
		Religiosity	0.01	0.03	[-0.05, 0.06]	0.01	0.849
		Contact Frequency	0.18	0.05	[0.09, 0.27]	0.19	< 0.001
**H2b**_**1**_	Bias ← Political orientation	(Intercept)	− 0.39	0.14	[-0.66, − 0.12]		0.005
		Political Orientation	0.08	0.03	[0.02, 0.13]	0.14	0.006
	With covariates	(Intercept)	0.08	0.29	[-0.49, 0.64]		0.783
		Political Orientation	0.1	0.03	[0.04, 0.16]	0.18	0.001
		Religiosity	0.02	0.03	[-0.03, 0.07]	0.04	0.471
		Contact Frequency	-0.13	0.04	[-0.21, − 0.04]	0.15	0.003
**H2b**_**2**_	SP allyship ← OP allyship + PO + OP allyship x PO	(Intercept)	5.29	0.04	[5.21, 5.38]		< 0.001
		OP Allyship	0.46	0.04	[0.38, 0.54]	0.48	< 0.001
		Political Orientation	0.21	0.02	[0.16, 0.26]	0.34	< 0.001
		OP Allyship x PO	-0.04	0.02	[-0.08, 0.01]	-0.07	0.065

### RQ1 – How is political orientation associated with allyship to the LGBTQ+ community?

#### H1a

Self-perceived allyship was regressed on political orientation (self-reported by cisgender, straight participants). Political orientation was a statistically significant predictor of self-perceived allyship to the LGBTQ+ community (*F*(1, 373) = 147.00, Adj. *R*^2^ = 0.28, *p* < .001). Specifically, cisgender, straight participants with more liberal political views tended to rate their own allyship more favorably than cisgender, straight participants with more conservative political views (*b* = 0.33, *p* < .001).

The overall model controlling for participants’ religiosity and frequency of contact between cisgender, straight participants and their LGBTQ+ counterparts was significant, *F*(3, 371) = 49.74, Adj *R*^2^ = 0.28, *p* < .001. In this model, political orientation still accounted for a large amount of unique variance in self-perceived allyship (*f*^2^ = 0.34).

#### H1b

Other-perceived allyship was regressed on political orientation (self-reported by cisgender, straight participants). Consistent with self-perceived allyship results, political orientation was a significant predictor of other-perceived allyship for the LGBTQ+ community (*F*(1, 373) = 69.08, Adj. *R*^2^ = 0.15, *p* < .001). More liberal (vs. conservative) cisgender, straight individuals tended to be rated as better allies by their LGBTQ+ close others (*b* = 0.25, *p* < .001). This relationship held after controlling for cisgender, straight and the frequency of contact between cisgender, straight participants and their LGBTQ+ identifying counterparts in the larger model, *F*(3, 371) = 28.98, Adj *R*^2^ = 0.18, *p* < .001. Political orientation continued to explain a moderate amount of unique variance in other-perceived allyship (*f*^2^ = 0.14).

Both H1b models violated the normality assumption for residuals (Shapiro-Wilk *W* = 0.98, *p* < .001). As a robustness check, we reran both using a quadratic transformation of the outcome variable (i.e., other-perceived allyship squared). Results were consistent with the untransformed results and are reported in the supplemental file.

### RQ2 – To what extent are there biases in perceptions of LGBTQ+ allyship among liberals and conservatives?

#### H2a

To evaluate self-other ratings alignment (vs. divergance), we calculated a difference score where positive values reflected overestimation and negative values reflected underestimation of allyship (i.e., bias; *M* = –0.03, *SD* = 0.94, range = − 3.55–4.09). Contrary to our expectations that there would be a general positivity bias, there was no evidence of a significant discrepancy, *t*(377) = –0.60, 95% CI [-0.12, 0.07], *p* = .550, *d* = –0.03. In general, results suggested there was no tendency for overestimations in self-perceptions of allyship among straight, cisgender individuals in this sample on average.

#### H2b

First, we evaluated whether political orientation was a statistically significant predictor of our calculated bias score. The overall model was significant (*F*(1, 373) = 7.79, Adj. *R*^2^ = 0.02, *p* = .006). More liberal (vs. conservative) cisgender, straight individuals tended to be positively biased in their evaluations of their allyship (*b* = 0.08, *p* = .006). The overall model accounting for religiosity and frequency of contact was also significant, *F*(3, 371) = 5.98, Adj. *R*^2^ = 0.04, *p* = .001. In this model, the influence of political orientation on bias remained a significant predictor of bias, albeit a small one (*f*^2^ = 0.03), indicating that liberals (vs. conservatives) were more likely to rate themselves as better allies than their LGBTQ+ close others did.

The residuals in the models described above violated normality (Shapiro-Wilk *W* = 0.97-0.98, *p* < .001). Results from the transformed version corresponded to our unadjusted results and are reported in the supplemental file.

**Fig. 1:**
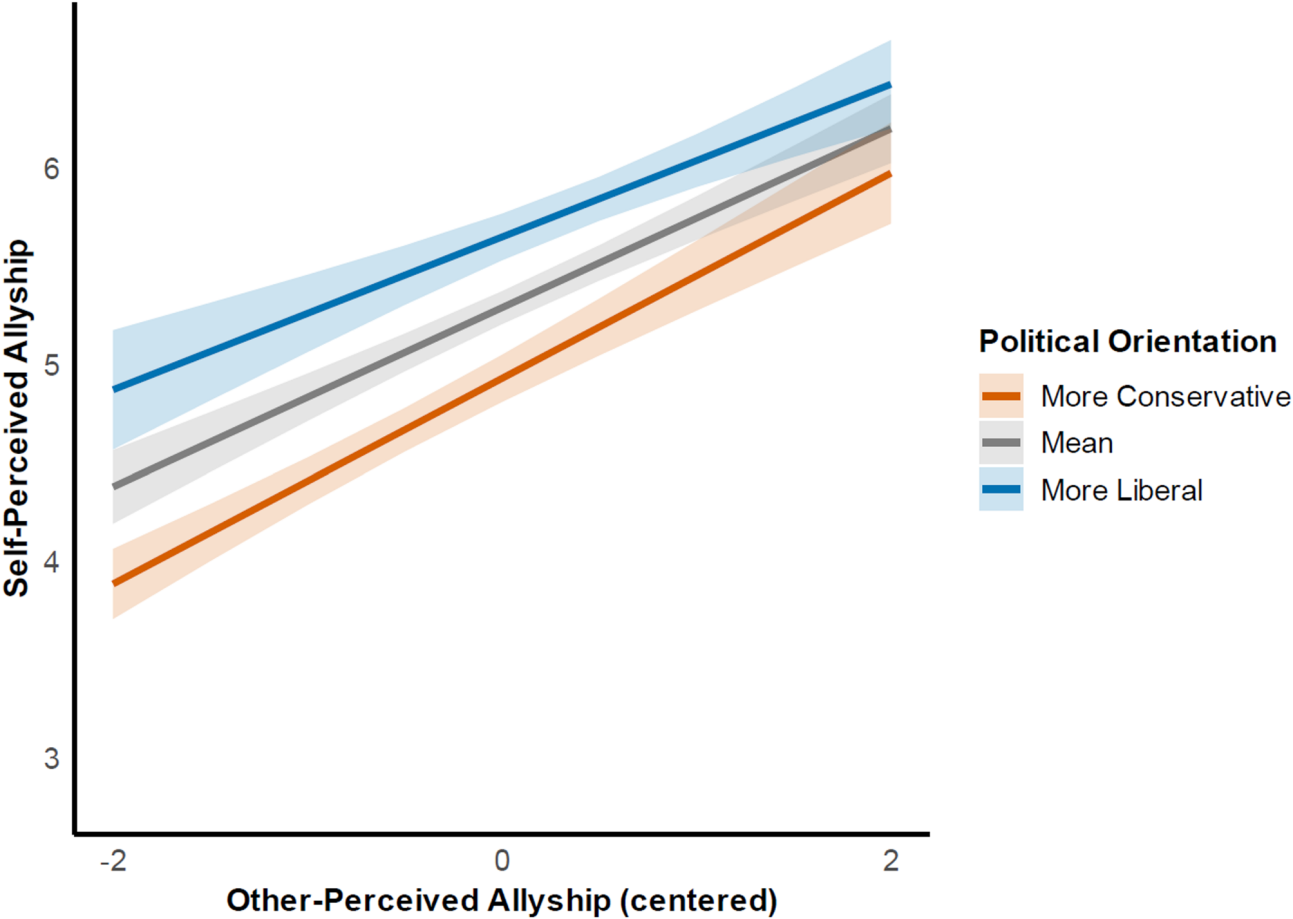
Self-perceptions of allyship as a function of political orientation and other-perceptions of allyship from LGBTQ+ close others. *Note*: Political orientation was mean-centered: Mean = 0, More Conservative = - 1 SD, More Liberal = +1 SD. Only the main effects of political orientation and other-perceived allyship were supported.

Next, we examined the extent to which other-perceived allyship and political orientation were influencing self-perceptions of allyship. Self-perceived allyship (rated by straight, cisgender respondents) was regressed on other-perceived allyship (rated by LGBTQ+ close others) and political orientation (self-reported by straight, cisgender respondents), and their interaction. Political orientation and other-perceived allyship were mean-centered prior to analysis. This model was significant, *F*(3, 371) = 117.60, Adj. *R*^2^ = 0.48, *p* < .001. Results suggested that both political orientation (*b* = 0.21, *p* < .001) and other-perceived allyship (*b* = 0.46, *p* < .001) were significant predictors of self-perceived allyship to the LGBTQ+ community (see Fig. 1). The interaction between political orientation and other-perceived allyship was not statistically significant (*b* = − 0.04, *p* = .065).

These results suggest that, even when rated equally in allyship by their LGBTQ+ close others, liberals tend to see themselves as better allies than conservatives. This finding supports our prediction that liberals may be more likely than conservatives to overestimate their allyship. Since the interaction term was not statistically significant, this effect appears consistent across different levels of perceived allyship from LGBTQ+ close others.

In addition, LGBTQ+ close other ratings (i.e., other-perceived allyship) may be a stronger predictor of self-perceived allyship than political orientation. Political orientation only explained a moderate amount of unique variance (*f*² = 0.19), whereas other-perceived allyship explained a large amount (*f*² = 0.40) in self-perceived allyship.

As the residuals in this model also violated normality (Shapiro-Wilk *W* = 0.98, *p* < .001), we reran the model using a quadratic transformation of the outcome. Results were consistent with the untransformed results and are reported in the supplemental file.

### Exploratory Analyses

We preregistered several exploratory analyses that did not have clear, directional hypotheses. We first examined whether components of allyship (non-prejudice, action, humility) were differentially influenced by political orientation and then examined how each component was associated with alignment (or divergence) in allyship ratings. Among the other exploratory analyses that were pre-registered, we report only those that revealed statistically significant effects that help to contextualize our main findings (EA1, EA3, and EA7), reported below. Full regression results for all exploratory analyses discussed in the text are presented in Table 5. Results for all other exploratory, pre-registered analyses are available in the supplemental file.

**Table 5:** Exploratory regression models and coefficients.

Exploratory Analysis	Outcome	Predictor	b	SE	95% CI	β	*p*	
**EA1 – Influence of age**	SP Allyship	(Intercept)	5.25	0.05	[5.16, 5.34]		< 0.001	
		Political Orientation	0.33	0.03	[0.28, 0.38]	0.54	< 0.001	
		Age	0	0	[–0.01, 0.01]	0.01	0.895	
		PO × Age	–0.01	0	[–0.01, –0.00]	–0.10	0.023	
	OP Allyship	(Intercept)	5.29	0.05	[5.19, 5.39]		< 0.001	
		Political Orientation	0.25	0.03	[0.19, 0.31]	0.39	< 0.001	
		Age	< 0.01	0	[–0.01, 0.01]	–0.02	0.666	
		PO × Age	< 0.01	0	[–0.01, 0.01]	< 0.01	0.986	
**EA3 – Influence of perceived closeness**	SP Allyship	(Intercept)	5.27	0.05	[5.18, 5.36]		< 0.001	
		Political Orientation	0.32	0.03	[0.27, 0.38]	0.53	< 0.001	
		Closeness	0.10	0.03	[0.04, 0.16]	0.14	< 0.001	
		PO x Closeness	− 0.07	0.02	[-0.10, − 0.03]	− 0.17	< 0.001	
	OP Allyship	(Intercept)	29.21	0.48	[28.26, 30.16]		< 0.001	
	*Transformed*	Political Orientation	2.32	0.28	[1.77, 2.87]	0.37	< 0.001	
		Closeness	2.31	0.31	[1.70, 2.93]	0.33	< 0.001	
		PO x Closeness	-0.31	0.18	[-0.66, 0.04]	− 0.08	0.081	
**EA7 – Implications for interpersonal trust**	Interpersonal Trust	(Intercept)	5.58	0.04	[5.50, 5.67]		< 0.001	
		Bias score	-0.21	0.05	[-0.30, − 0.11]	-0.22	< 0.001	
	Interpersonal Trust	(Intercept)	5.57	0.05	[5.48, 5.66]		< 0.001	
		OP Allyship	0.36	0.05	[0.26, 0.45]	0.45	< 0.001	
		SP Allyship	-0.03	0.05	[-0.13, 0.06]	-0.04	0.488	
		SP Allyship x OP Allyship	0.03	0.03	[-0.03, 0.08]	0.04	0.389	

### Components of allyship

Research^6^ has identified three related components of allyship to the LGBTQ+ community: (1) non-prejudice toward LGBTQ+ individuals, (2) action in support of LGBTQ+ rights, and (3) humility when discussing LGBTQ+ issues. We examined whether political orientation is more strongly associated with any of these dimensions. Table 6 presents bivariate correlations between these allyship components (both self- and other-perceived) and key variables.

**Table 6:** Means, standard deviations, and correlations for allyship components (non-prejudice, action, and humility) with confidence intervals.

Variable	M	SD	1	2	3	4	5	6	7	8	9
1. SP Political Orientation	4.64	1.74									
2. OP Political Orientation	4.85	1.83	0.82***								
			[0.78, 0.85]								
3. SP Non-prejudice	6.13	0.98	0.44***	0.36***							
			[0.36, 0.52]	[0.27, 0.44]							
4. SP Action	4.62	1.78	0.60***	0.50***	0.53***						
			[0.53, 0.66]	[0.42, 0.57]	[0.46, 0.60]						
5. SP Humility	4.94	1.43	0.16**	0.10	0.18**	0.31***					
			[0.06, 0.26]	[-0.00, 0.20]	[0.08, 0.28]	[0.21, 0.40]					
6. OP Non-prejudice	6.03	1.00	0.37***	0.39***	0.54***	0.42***	0.17***				
			[0.28, 0.49]	[0.30, 0.47]	[0.46, 0.61]	[0.33, 0.50]	[0.07, 0.27]				
7. OP Action	4.75	1.57	0.41***	0.44***	0.40***	0.64***	0.29***	0.59***			
			[0.32, 0.49]	[0.35, 0.52]	[0.32, 0.49]	[0.58, 0.70]	[0.19, 0.38]	[0.52, 0.65]			
8. OP Humility	5.12	1.28	0.13*	0.16**	0.16**	0.22***	0.41***	0.40***	0.48***		
			[0.03, 0.23]	[0.06, 0.26]	[0.06, 0.26]	[0.13, 0.32]	[0.33, 0.49]	[0.31, 0.48]	[0.40, 0.56]		
9. Religiosity	3.58	1.90	− 0.36***	− 0.30***	− 0.17***	− 0.18***	0.01	− 0.16**	− 0.14**	-0.07	
			[-0.45, − 0.27]	[-0.39, − 0.21]	[-0.27, − 0.07]	[-0.28, − 0.08]	[-0.09, 0.11]	[-0.26, − 0.06]	[-0.23, − 0.04]	[-0.17, 0.03]	
10. Frequency of Contact	4.95	1.12	0.16**	0.17***	0.11*	0.11*	0.00	0.19***	0.22***	0.17***	− 0.15**
			[0.06, 0.26]	[0.07, 0.27]	[0.00, 0.20]	[0.01, 0.21]	[-0.10, 0.10]	[0.09, 0.29]	[0.12, 0.32]	[0.07, 0.27]	[-0.25, − 0.05]

Notably, political orientation showed a weaker correlation with self-rated humility (*r* = .16, *p* = .002) compared to non-prejudice (*r* = .44, *p* < .001) and action (*r* = .60, *p* < .001). This pattern also held for LGBTQ+ participants’ perceptions of their cisgender, straight close others. These results suggest that self- and other-perceptions of non-prejudice and action may be more aligned with political orientation than perceptions of humility.

We also explored whether these associations could explain the small but significant tendency for liberals to rate themselves higher on allyship than conservatives, controlling for other-perceived allyship. As in RQ2, we regressed self-perceived allyship on others’ perceptions of allyship, political orientation, and their interaction, running separate models for each component of allyship.

Results indicated that, although political orientation significantly predicted self-perceptions for all three aspects of allyship, the relationship was strongest for action (*b* = .41, *p* < .001) relative to non-prejudice (*b* = .15, *p* < .001) or humility (*b* = .09, *p* = .022). These findings suggest that liberals’ (vs. conservatives’) tendency to rate themselves as better allies may be particularly driven by discrepancies in perceived action on behalf of the LGBTQ+ community. Full model results are reported in the supplemental file.

### EA1 – Influence of age

We explored whether age of the cisgender, straight participants moderated the relationship between political orientation and allyship (both self-perceived and other-perceived). We suspected that because younger (vs. older) generations may have a more inclusive stance regarding LGBTQ+ people considering greater identification^1^ and institutional changes supporting LGBTQ+ people (e.g., same-sex marriage legalization^43)^, that the relationship between political orientation and allyship may be weaker for younger (vs. older) potential allies. Allyship (self-perceived and other-perceived) was regressed onto cisgender, straight participant age, political orientation (self-reported by cisgender, straight participants), and their interaction. Political orientation and age were mean-centered prior to analysis. Any statistically significant interactions were probed at high (+ 1 SD) and low (-1 SD) levels for age.

**Self-perceived allyship.** The overall model was statistically significant (*F*(3, 371) = 51.16, Adj. *R*^2^ = 0.29, *p* < .001). Political orientation, but not age, was a statistically significant predictor of self-perceived allyship for the LGBTQ+ community. There was also a significant interaction between political orientation and age predicting self-perceived allyship to the LGBTQ+ community (*b* = − 0.01, *p* = .023), suggesting that the influence of political orientation on self-perceptions of allyship may depend on the potential ally’s age. Contrary to our expectations, simple slopes indicated that the relationship between political orientation and self-perceived allyship was stronger among younger cisgender, straight participants (*b* = 0.40, 95% CI [0.32, 0.48], *SE* = 0.04, *t* = 9.76, *p* < .001), with the relationship weaker among older cisgender, straight participants (*b* = 0.27, 95% CI [0.19, 0.34], *SE* = 0.04, *t* = 7.04, *p* < .001), relative to the relationship for average-age cisgender, straight participants (*b* = 0.33, 95% CI [0.28, 0.38], *SE* = 0.03, *t* = 12.28, *p* < .001).

**Other-perceived allyship.** The overall model was significant (*F*(3, 371) = 22.98, Adj. *R*^2^ = 0.15, *p* < .001). Results revealed only political orientation as a significant predictor of other-perceived allyship to the LGBTQ+ community. Results did not support an interaction between political orientation and age (*b* < 0.01, *p* = .986) or an individual influence of age predicting other-perceptions of allyship toward the LGBTQ+ community. Taken together with the results for self-perceived allyship, these findings suggest that age may predict how cisgender, straight individuals evaluate their own allyship behaviors, but it is not associated with how they are perceived by their LGBTQ+ close others.

This model violated the assumption that residuals are normally distributed (Shapiro-Wilk *W* = 0.98, *p* < .001), therefore, we employed a quadratic transformation of the outcome variable (i.e., other-perceived allyship squared). Transformed results mirrored the untransformed results and reported in the supplemental file.

### EA3 – Influence of perceived closeness

We explored whether the relationship between political orientation and allyship depended on the level of closeness between straight, cisgender individuals and their LGBTQ+ identifying counterparts. We expected that the relationship between political orientation and allyship may be weaker for dyads who have higher (vs. lower) levels of perceived closeness. Allyship (self-perceived and other-perceived) was regressed on perceived closenesss (self-reported by LGBTQ+ participants), political orientation (self-reported by cisgender, straight participants), and their interaction. Political orientation and perceived closeness were mean-centered prior to analysis. Any statistically significant interactions were probed at high (+ 1 SD) and low (-1 SD) levels for perceived closeness.

**Self-perceived allyship.** The overall model was statistically significant (*F*(3, 371) = 62.79, Adj. *R*^2^ = 0.33, *p* < .001). Political orientation and perceived closeness were statistically significant predictors of self-perceived allyship to the LGBTQ+ community in this model. These effects were qualified by a significant interaction between political orientation and perceived closeness predicting self-perceived allyship to the LGBTQ+ community, suggesting that the influence of political orientation on self-perceptions of allyship may depend on the level of closeness in the relationship. Consistent with our expectations, the relationship between political orientation and self-perceived allyship was weaker when perceived closeness was high (*b* = 0.22, 95% CI [0.15, 0.29], *SE* = 0.04, *t* = 6.23, *p* < .001), with the relationship stronger when perceived closeness was low (*b* = 0.43, 95% CI [0.35, 0.50] *SE* = 0.04, *t* = 11.38, *p* < .001). The political orientation and self-perceived allyship relationship for average closeness fell in the middle (*b* = 0.32, 95% CI [0.27, 0.38], *SE* = 0.03, *t* = 12.47, *p* < .001).

Residuals were not normally distributed in this model (Shapiro-Wilk *W* = 0.99, *p* = .006). We employed a quadratic transformation of the outcome variable (i.e., self-perceived allyship squared) and results were consistent with the untransformed results reported and are included in the supplemental file.

**Other-perceived allyship.** The overall model was statistically significant (*F*(3, 371) = 49.07, Adj. *R*^2^ = 0.28, *p* < .001). Political orientation and perceived closeness were statistically significant predictors of other-perceived allyship to the LGBTQ+ community in this model. Consistent with the results for self-perceptions of allyship, results indicated an interaction between political orientation and perceived closeness predicting other-perceived allyship to the LGBTQ+ close other, suggesting that the influence of political orientation on other-perceptions of allyship may depend on the degree of closeness. However, after applying a quadratic transformation to the outcome variable (i.e., other-perceived allyship squared) in response to non-normally distributed residuals (Shapiro-Wilk W = 0.99, *p* = .002), the results diverged from those obtained using the untransformed data. Specifically, the interaction term was no longer statistically significant (*p* = .081). Given this deviation, we align with the transformed results interpretation, which are reported in full in Table 5. These results show only statistically significant main effects of political orientation and perceived closeness on other-perceived allyship. That is, allyship was perceived more positively both among cisgender, straight individuals who were more politically liberal (vs. conservative) and among those who shared a closer (vs. more distant) relationship with the LGBTQ+ perceiver.

### EA7 – Implications for Interpersonal trust

We examined whether the discrepancy between self- and other-perceptions of allyship influenced perceptions of interpersonal trust as reported by LGBTQ+ individuals. We wondered whether overestimation of allyship by cisgender, straight close others might undermine interpersonal trust, whereas underestimation (i.e., a humility bias) might enhance it. We also considered the possibility that greater alignment (i.e., matching) between self- and other-perceptions of allyship would predict higher levels of interpersonal trust.

The overall model was significant (*F*(1, 376) = 19.60, Adj. *R*^2^ = 0.05, *p* < .001. There was a negative influence of bias in allyship predicting interpersonal trust (*b* = − 0.21, *p* < .001), such that the tendency to overestimate ones’ allyship was associated with reduced interpersonal trust as reported by the LGBTQ+ identifying close other. This finding was consistent with the idea that overestimations of allyship would be associated with a decrease in interpersonal trust.

We recognize that difference scores may not be considered best practice for examining questions of agreement and disagreement^42,52,53^. Therefore, we used a different model to probe this question further. We regressed interpersonal trust on other-perceived allyship (mean-centered), self-perceived allyship (mean-centered), and their interaction. The overall model was significant (*F*(3, 374) = 25.74, Adj. *R*^2^ = 0.16, *p* < .001). Results supported only a statistically significant influence of other-perceived allyship ratings on interpersonal trust (*b* = 0.36, *p* < .001). Self-perceived allyship and the interaction between self-perceived and other-perceived allyship were not statistically significant predictors (*p*s > 0.05). In conjunction with the results of the model that reduced the self-other ratings into a difference score, results support the idea that how a potential ally is *perceived* may bear the greatest importance on the LGBTQ+ group members sense of interpersonal trust, regardless of how a potential ally rates themselves on allyship behaviors.

Residuals were not normally distributed in these models (Shapiro-Wilk *W* = 0.98-0.99, *p* < .01). We employed a quadratic transformation of the outcome variable (i.e., interpersonal trust squared). Results were consistent with the untransformed results and are reported in full in the supplemental file.

## Discussion

In this study, we examined the relationship between political orientation and allyship to the LGBTQ+ community, considering both self-perceptions from potential allies as well as other-perceptions from LGBTQ+ close others in their social network.

Our findings supported our hypotheses that political orientation is associated with allyship to the LGBTQ+ community. Specifically, individuals who identified as more liberal (as opposed to conservative) reported higher levels of allyship and were also perceived as better allies by members of the LGBTQ+ community who knew them well. This pattern aligns with prior research showing that liberals tend to express more positive attitudes toward marginalized groups^22,33^, endorse egalitarian values more strongly^17,18^, and report greater intentions for collective action^14,20^. Although general support for LGBTQ+ rights has increased over time^43^(especially in regions with supportive institutional changes^54)^, polling indicates that as of 2025, conservatives’ attitudes toward LGBTQ+ rights have become less positive since peaking in 2021[55]—a trend consistent with our findings.

We further examined whether potential allies’ self-perceptions of allyship aligned (or diverged) with how they were perceived by LGBTQ+ close others. Overall, we found strong congruence between self- and other-perceptions of allyship. This pattern—evidenced by small differences between self- and other-perceptions—may run counter to typical self-enhancement biases^34^ but is consistent with prior research demonstrating a strong correlation between self- and other-evaluations of allyship from LGBTQ+ cohabitants [6 ] .

We also found preliminary evidence that liberals may exhibit bias in their self-assessments of allyship. Both other-perceived allyship and straight, cisgender political orientation were unique and significant predictors of self-perceived allyship. In other words, when accounting for how allyship behaviors were perceived by LGBTQ+ close others, those who identified as more liberal continued to rate themselves as better allies than their conservative counterparts, though the effect size was small. This small, systematic bias may stem from the overlap between political identity and group affiliation. That is, liberals may experience greater psychological overlap with the LGBTQ+ community [19 ] and are generally more likely to endorse egalitarian beliefs than conservatives^17,18^. For cisgender, straight liberals, allyship may serve as both a reflection of their political identity and a source of self-worth. Thus, liberals may be especially motivated to view themselves positively in this domain, as allyship could be tied to their self-esteem and self-image^34,56^. It is also possible that this tendency for overestimation may be reflective of performative allyship^41^, if it is the case that personal motivations^14^ for seeing oneself as a “good” ally incentivize liberals (vs. conservatives) to overestimate their allyship behaviors relative to how they are viewed by LGBTQ+ individuals. While we conducted exploratory analyses that examined self- and other-perceived motives for allyship, they were inconclusive (see our supplemental file) and warrant future study.

Our exploratory analyses revealed additional information regarding factors related to allyship perceptions. We identified higher interpersonal trust as experienced by LGBTQ+ individuals as an outcome of their greater perception of allyship behaviors of their cisgender, straight close others. While our data suggest that self-perceptions largely map onto other-perceptions, self-perceptions of allyship were not predictive of interpersonal trust when accounting for other-perceptions of allyship. Therefore, it seems that LGBTQ+ close others’ perceptions may be more associated with interpersonal trust rather than the degree of matching between self- and other-perceptions. Our findings are consistent with studies in other intergroup contexts showing that greater perceptions of allyship lead to greater feelings of trust and belonging^57,58^. Indeed, perceived or true allyship, rather than self-proclaimed allyship, may be one pathway to cultivating positive relationship outcomes in cross-group interactions.

We conducted follow-up analyses to examine the specific components of allyship, non-prejudice, action, and humility, to determine whether particular components were more (or less) strongly associated with political orientation. Among these components, action showed the strongest correlation with political orientation, whereas humility exhibited the weakest. The relatively stronger association between political orientation and action may reflect liberals’ (vs. conservatives’) greater inclination toward collective action^20,14^. Further, additional exploratory analyses indicated that the difference between liberal and conservative self-perceptions of allyship (after controlling for other-perceptions of allyship) seemed most pronounced in the action component, suggesting that liberals (vs. conservatives) may be especially likely to view themselves as more active in LGBTQ+ advocacy than they are perceived to be by their LGBTQ+ close others. Importantly, because an *action orientation* may be a key criterion by which members of the LGBTQ+ community evaluate potential allies, accurately perceiving one’s own behavior may be an essential step to becoming a more effective ally^9^.

Humility, or the tendency for cisgender, straight individuals to decenter themselves in conversations about LGBTQ+ issues and prioritize listening to LGBTQ+ voices over sharing their own opinions, was the component of allyship least strongly associated with political orientation. We reason that individuals across the political spectrum could adopt humility, attenuating the association between liberalism and humility. Broadly, cisgender, straight individuals may experience discomfort when discussing LGBTQ+ issues in intergroup contexts, potentially due to concerns about being perceived as prejudiced^59^. Among liberals, higher levels of intergroup contact^21^ may raise awareness of the limits of one’s own perspective and foster an affiliative motivation. In contrast, lower levels of contact among conservatives may contribute to reduced comfort engaging in LGBTQ+-focused conversations, reflecting a more avoidance-based motivation. In turn, humility may carry different meanings for liberals and conservatives, suggesting a combination of concerns about negative social evaluations and heightened awareness of LGBTQ+ perspectives.

Individual and relational factors also may shape how allyship is evaluated. In particular, we explored whether the association between political orientation and allyship to the LGBTQ+ community varied as a function of straight, cisgender individuals’ age. Results indicated that the association between political orientation and self-perceived allyship was strongest among younger potential allies and weakest among older potential allies. This finding was contrary to our expectation that the relationship would be stronger among older adults, given younger adults’ greater likelihood of interpersonal contact with LGBTQ+ individuals^29^ and the level of identification with the LGBTQ+ community in younger (vs. older) age cohorts^1^. We interpreted these generational trends as indicative of a generally higher baseline of allyship behaviors among younger adults, irrespective of political orientation.

One possible explanation for our findings is that younger cisgender, straight individuals may have stronger *partisan identities*^60,61^ than older cisgender, straight individuals, thereby amplifying the link between political orientation and self-perceived allyship to the LGBTQ+ community. However, research supports the opposite: affective polarization tends to increase with age^62^. It is possible that other factors related to partisan identity, such as ideological sorting^63^, as well as broader period and cohort effects^62^, may have unique and unclear implications for the age-related patterns we observed. Younger adults may be coming of age in an increasingly polarized political climate^64^, and this context may serve as a salient reference point in shaping their self-evaluations of allyship. Regardless, when considering other-perceptions of allyship, age no longer functioned as a significant moderator. This pattern suggests that certain individual characteristics, such as age, may be associated with self-perceptions of allyship, but not with how one’s allyship is perceived by members of the community it is intended to support.

In our data, potential allies may view closeness as a central factor in their self-assessments of allyship, potentially diminishing the degree of association between allyship and political orientation. In contrast, LGBTQ+ perceivers may be more likely to consider closeness as one of several distinct and independent factors (alongside variables like political orientation) that are related to their overall judgements of allyship. These findings are consistent with prior research indicating that greater closeness to LGBTQ+ individuals predicts greater propensity to engage in allyship^30^. Our findings also extend emerging scholarship on how evaluations of allyship behaviors should consider relational closeness. For example, minor actions (e.g., wearing a pride flag pin) may carry more significance when performed by a new acquaintance than by a close friend or family member^9^.

### Implications

The results of this study carry several important implications. Political orientation appears to be a meaningful individual-level predictor of allyship to the LGBTQ+ community and may also reflect broader mechanisms underlying social change efforts. Previous research has suggested that motivations for straight, cisgender individuals’ allyship can stem from belief-based factors (e.g., moral convictions^13,14)^ as well as interpersonal experiences (e.g., close relationships with LGBTQ+ individuals^30,65^) . Our findings align with this work, demonstrating that both political orientation and relational closeness are associated with allyship to the LGBTQ+ community.

Recognizing the link between political orientation and allyship highlights the importance of addressing the “political elephant in the room.” Trainings, workshops, and other allyship-promoting interventions may be perceived as politically liberal^66^, which may engender a resistance among ideologically conservative participants^67^. If the goal of interventions is to foster allyship among a broader range of cisgender, straight individuals, it is critical to acknowledge that people may be starting from different ideological positions. As such, interventions may need to be tailored accordingly. For instance, reducing the political framing of allyship may be more effective for engaging conservatives^68^, whereas explicitly connecting allyship to political identity may enhance its salience and motivational value for liberals. Developing ideologically sensitive approaches could help ensure that allyship efforts are inclusive, effective, and sustainable across the political spectrum.

Beyond its implications for understanding the role of political orientation in allyship behaviors, our research also offers important insights for close relationships. First, our findings suggest that self-perceptions of allyship among cisgender, straight individuals are generally accurate, aligning with how they are perceived by their LGBTQ+ close others. Further, while one’s political orientation and perceived level of allyship may not determine the formation or existence of the kinds of cross-group relationships reflected in our sample, they may still be related to key relational outcomes, such as interpersonal trust. Prior research supports this notion that perceiving allyship from cisgender, straight individuals is positively associated with other important aspects of relationship functioning, including greater feelings of authenticity^10^ and better relationship quality^6^. Collectively, these findings highlight the potential interpersonal benefits of allyship and suggest that more positive perceptions of it may enhance the quality of such cross-group relationships.

### Limitations and future directions

Several limitations of the present study warrant consideration. First, the correlational nature of our design limits the conclusions we can draw from our findings, as we cannot infer causality or directionality. Although prior research suggests that more liberal (vs. conservative) individuals may be more motivated to engage in allyship efforts^13^, it is also possible that engaging in allyship leads individuals to adopt more liberal (vs. conservative) political leanings. Thus, liberalism and LGBTQ+ allyship may have a bidirectional relationship: becoming more liberal may align individuals with causes such as LGBTQ+ rights advancement, while becoming an LGBTQ+ ally may motivate broader participation in liberal activism. Our other findings are similarly open to alternative interpretations. For example, individuals who identify as LGBTQ+ allies may assume greater closeness to LGBTQ+ people in their social circle, just as becoming close with LGBTQ+ people may motivate allyship behaviors. Future research should use alternative designs to clarify the directionality of these relationships and better understand the antecedents and consequences of allyship.

Our design also focused on close others, many of which involved friendships or cohabitating individuals at the time of data collection. While this context provides insight into intimate, real-world relationships, it may limit the generalizability of our findings to other types of relationships. Future research should consider alternative paradigms that prioritize less intimate or more contextually diverse relationships (e.g., those in workplace or educational settings) to better understand how political orientation influences allyship to the LGBTQ+ community across different social environments.

An additional limitation of the current study concerns our measurement of contact. Rather than assessing general frequency of contact with members of the LGBTQ+ community, we focused specifically on the frequency of contact within each dyad. This measure of contact was included as a covariate in our primary analyses. Frequency of contact with members of the target group has been a central focus in prior research on intergroup relations and collective action^16,31,69^. Other work has also suggested that the *quality* of contact—the extent it is perceived as positive or negative—may be a powerful predictor of attitudes toward the outgroup and subsequent social action on their behalf^32^. Moreover, research also emphasizes the importance of accounting for factors that can shape the influence of intergroup contact on social change (see for review^70^). Such factors include perceived legitimacy of group-based disparities^71^, advantaged group members’ propensity to adopt inclusive social identities^72^, and the extent to which members of the target group are perceived to be affected by bias^73^. Future research should more rigorously investigate both the frequency *and* quality of contact, as well as relevant contextual moderators, to arrive at a better understanding of how these dynamics interact with political orientation and allyship to the LGBTQ+ community.

Another limitation of the present study concerns the scope of our allyship measure, which assessed allyship to the LGBTQ+ community as a whole. While this inclusive approach is valuable, it may obscure important variability in allyship tendencies toward specific subgroups (e.g., sexual minorities vs. gender minorities). It is possible that political orientation differentially predicts allyship depending on the specific group in question, especially in light of political discourse and policy debates surrounding transgender rights in recent years^24,74^. Future research should explore whether political orientation has distinct associations with allyship toward different segments of the LGBTQ+ community.

Relatedly, while our findings contribute to the growing literature on LGBTQ+ allyship, they may not generalize to other forms of allyship, such as allyship to people of color, women, or individuals with disabilities. Future work should investigate whether the patterns observed here, particularly regarding the role of political orientation, extend to allyship in other intergroup contexts.

Finally, one statistical limitation of our study is the use of difference scores in our analysis strategy. Difference scores have been criticized for several reasons, including their tendency to reduce variability^52^ and their limited precision in capturing the nuances of matched perceptions^53^. While more sophisticated statistical techniques commonly used in dyadic research, such as the Truth and Bias Model^42^ or Response Surface Analysis^75^, can address these limitations, they were unfortunately not well-suited to the structure of our data. We had initially planned to use the Truth and Bias Model as outlined in our pre-registration. However, during data analysis, we determined that this model was not appropriate for our research question due to its asymmetry: the key variable of interest (allyship) applied only to one member of the dyad (the cisgender, straight close other). Our study did not include an equivalent measure of allyship for LGBTQ+ participants (e.g., advocacy or solidarity for members of their own community), which made it incompatible with the assumptions of the Truth and Bias framework. Future research could address the limitations of these data and consider alternative designs or measures that more fully capture the complexity of dyadic perception data on allyship to the LGBTQ+ community.

## Conclusion

This study investigated the relationship between political orientation and allyship to the LGBTQ+ community, drawing from both self-reports from cisgender, straight individuals and perceptions from LGBTQ+ individuals within their social circles. Results revealed a strong association: cisgender, straight individuals who identified as more liberal (compared to conservative) were more likely to both view themselves and be viewed by LGBTQ+ others as better allies. Although cisgender, straight individuals were generally accurate in their self-perceptions of allyship, liberals exhibited a small bias in overestimating their levels of allyship, relative to conservatives. Exploratory analyses also revealed that interpersonal trust may be more strongly associated with other-perceptions than self-perceptions of allyship, supporting the idea that it is true allyship, as defined by LGBTQ+ people, that is associated with positive interpersonal outcomes. Together, these findings highlight political orientation as meaningfully associated with LGBTQ+ allyship and contribute to a broader understanding of the factors that are related to social support and action on behalf of the LGBTQ+ community.

## Electronic Supplementary Material

Below is the link to the electronic supplementary material.

Supplementary Material 1

## Data Availability

The dataset supporting the conclusions of this article is available on OSF at https://osf.io/2q7w6/overview?view_only=b9f680082390416a90c3e456aeee29b9. Raw data containing personally identifiable information cannot be shared due to participant privacy and ethical restrictions. The shared dataset includes all dyads that passed preregistered exclusion criteria, with anonymized dyad IDs and all variables used in the analyses. Also included on OSF are our “read me” file and study materials.

## Code Availability

Code for all analyses can be found here: https://osf.io/2q7w6/overview?view_only=b9f680082390416a90c3e456aeee29b9.

## Declarations

***Protocol Registration***.

The protocol, as accepted by the journal, can be found at: https://osf.io/2q7w6/?view_only=b9f680082390416a90c3e456aeee29b9.

***Correspondence*** and requests for materials should be addressed to H.B.

### Reprints and permissions information

is available at www.nature.com/reprints.
