# Supplementary material for: Associations between political orientation and allyship: Evidence from potential allies and their LGBTQ+ close others
Source: Sci Rep. 2026 Mar 1;16:8879. doi: 10.1038/s41598-026-42213-8 (PMC12988167; doi:10.1038/s41598-026-42213-8)
Supplement: Supplementary file 1 — Supplementary Material 1 [file 41598_2026_42213_MOESM1_ESM.pdf]

Table of Contents

Supplemental sample data-- 2

Transformed results for RQ1 and RQ2--- 3

Exploratory analyses (EAs)--5

Phase of recruitment--- 5

Components of Allyship--8

EA1 - Age--10

EA2 - Social and economic conservatism---10

EA3 - Closeness--12

EA4 - Authenticity--- 13

EA5 - LGBTQ+ identity centrality-- 15

EA6 - Internal and external motivations-- 16

EA7 - Interpersonal trust--- 18

EA8 - Allyship norm-- 18

Supplemental sample data

Table S1

Relationship dynamics of all dyads

Category	% (*n*)
Relationship Type	
Friend	55.7% (210)
Family	26.0% (98)
Co-worker	6.4% (24)
Significant Other	6.1% (23)
Acquaintance	3.4% (13)
Roommate	2.4% (9)
Cohabiting	
Yes	76.3% (212)
No	23.7% (66)

Note. Relationship type was only recorded as part of the survey for LGBTQ+ participants and was measured using the item, "Which of the following best describes how you know [nominee/person who nominated you]?" Percentages for relationship type were taken out of total responses (n = 377). The cohabiting measure was added to the survey during data collection and had 278 total responses.

Exclusion of Republicans and Democrats due to exclusion criteria

Among cisgender, straight participants, 8.61% of self-identified Republicans and 10.13% of self-identified Democrats were dropped following attention check exclusions. Excluding participants who did not form a complete dyad removed 53.26% of Republicans and 42.38% of Democrats. Excluding possible self-nominators reduced the share of Republicans by 10.08% and Democrats by 5.43%.

Transformed Results for RQ1 and RQ2

H1b

Both models violated the normality assumption for residuals (Shapiro-Wilk $W = .98$, $p < .001$). We reran both using a quadratic transformation of the outcome variable (i.e., other-perceived allyship squared). Overall, Models 1a ($F(1, 373) = 66.25$, Adj. $R^2 = .15$, $p < .001$) and 1b ($F(3, 371) = 28.02$, Adj. $R^2 = .18$, $p < .001$) were statistically significant. See Table S2 for full model results.

Table S2

H1b Transformed Results

Models	Predictor	*b*	*SE*	*p*	Adj. R^2
**Model 1a: (Other-perceived Allyship)^2 ← Political orientation**					
	(Intercept)	17.88	1.48	< .001	
	Political Orientation	2.43	0.30	< .001	.15
**Model 1b: With covariates**					
	(Intercept)	10.18	3.08	.001	
	Political Orientation	2.22	0.32	< .001	
	Religiosity	-.05	0.29	.851	
	Frequency of Contact	1.79	0.46	< .001	
					.18

Note. All predictors were centered at their mean.

H2b

The residuals in both models predicting bias violated normality (Shapiro-Wilk $W = .97$-$.98$, $p < .001$). We tried a square-root transformation of the outcome variable, bias, but the residuals were still not normally distributed. Instead, a robust data transformation was performed using M-estimators (Huber weights), which downweight extreme cases in a dataset

(see OSF for code used to conduct this robust data transformation; https://osf.io/2q7w6/overview?view_only=b9f680082390416a90c3e456aeee29b9). The parameter Phi (ϕ) was adjusted iteratively, based on results of multivariate normality (i.e., multivariate kurtosis). After multivariate normality was satisfied (b2p = 22.83, kurtosis = -1.64, p = .100), the null hypothesis was retained at ϕ of .02. After re-running the analyses with the newly transformed data, both Models 2a ($F(1, 373) = 9.77$, Adj. $R^2 = .02$, $p = .002$) and 2b ($F(3, 371) = 6.76$, Adj. $R^2 = .04$, $p < .001$) were statistically significant.

The overall model predicting self-perceived allyship also showed that residuals violated normality (Shapiro-Wilk $W = .98$, $p < .001$). We reran the model using a quadratic transformation of the outcome. This model (Model 3) was statistically significant ($F(3, 371) = 111.30$, Adj. $R^2 = .47$, $p < .001$). See Table S3 for full model results.

Table S3
H2b Transformed Results

Models	Predictor	*b*	*SE*	*p*	Adj. R^2
**Model 2a: Bias ←Political orientation**					
	(Intercept)	-.42	.13	.001	
	Political Orientation	.08	0.03	.002	.02
**Model 2b: With covariates**					
	(Intercept)	-.04	.28	.887	
	Political Orientation	.11	0.03	< .001	
	Religiosity	.03	0.03	.286	
	Frequency of Contact	-.12	0.04	.004	
					.04
**Model 3: (Self-perceived allyship)^2 ← Political orientation x Other-perceived Allyship**					
	(Intercept)	28.91	.43	< .001	
	Political Orientation	2.00	0.25	< .001	

Table S3

H2b Transformed Results

Models	Predictor	*b*	*SE*	*p*	Adj. R^2
	Other-perceived Allyship	4.69	0.41	< .001	
	Political Orientation x Other-perceived Allyship	-.08	0.21	.698	
					.47

Note. All predictors were centered at their mean.

Exploratory analyses

Exploratory analyses are grouped by type of analysis. Table S4-6 includes results for all regression models run.

Phase of recruitment

We explored whether the recruitment method influenced the relationship between political orientation and allyship by examining recruitment as a covariate. Phase of recruitment was a dichotomous variable (0 = Phase 1, 1 = Phase 2), reflecting whether participants were recruited directly online (Phase 1) or were nominated by the Phase 1 participants (Phase 2). Allyship (self-perceived, other-perceived) was regressed onto political orientation and phase of recruitment.

Self-perceived allyship

The model was statistically significant ($F(2, 372) = 73.30$, Adj. $R^2 = .28$, $p < .001$). Phase of recruitment was not a statistically significant predictor of self-perceived allyship for the LGBTQ+ community ($b = .02$, $p = .872$). Political orientation remained a statistically significant predictor of self-perceived allyship. Full regression results are reported in Table S4.

Other-perceived allyship

The overall model was statistically significant ($F(2, 372) = 37.97$, Adj. $R^2 = .17$, $p < .001$). Both political orientation and recruitment phase significantly predicted other-perceived allyship. Indeed, LGBTQ+ participants who were nominated by a cisgender, straight close other tended to rate that person's allyship more positively than participants who were recruited directly online ($b = .26$, $p = .015$), after accounting for political orientation.

The residuals in this model also violated normality (Shapiro-Wilk $W = .98$, $p < .001$), so we reran the model using a quadratic transformation of the outcome. Results were consistent with the untransformed results and are reported in Table S4.

Table S4

Regression results for EA1 – EA3

Models	Outcome	Predictor	*b*	*SE*	*p*	Adj. R^2
**Phase of Recruitment**	SP Allyship	(Intercept)	3.75	.13	< .001	
		Political Orientation	.32	.03	< .001	
		Phase	.02	.10	.872	.28
	OP Allyship	(Intercept)	3.91	.17	< .001	
		Political Orientation	.27	.03	< .001	
		Phase	.26	.11	.015	.17
**EA1 - Age**	**OP Allyship - Transformed**	(Intercept)	2.92	0.52	< .001	
		Political Orientation	2.42	0.30	< .001	
		Age	-.01	0.05	0.762	
		PO x Age	< -.01	0.03	0.999	0.14
**EA2 - Social Conservatism**	**SP Allyship**	(Intercept)	5.29	0.05	< .001	

		Political Orientation	0.27	0.04	< .001	
		Social Conservatism	-.01	.00	0.031	
		PO x SC	< .01	.00	0.402	0.29
	**OP Allyship**	(Intercept)	5.26	0.06	< .001	
		Political Orientation	0.22	0.04	< .001	
		Social Conservatism	< -.01	.00	0.200	
		PO x SC	< -.01	.00	0.269	0.16
	**OP Allyship - Transformed**	(Intercept)	28.7	0.61	< .001	
		Political Orientation	2.12	0.39	< .001	
		Social Conservatism	-.05	0.03	0.104	
		PO x SC	-.02	0.01	0.129	0.16
**EA2 - Economic Conservatism**	**SP Allyship**	(Intercept)	5.28	0.05	< .001	
		Political Orientation	0.26	0.03	< .001	
		Economic Conservatism	-.01	.00	0.007	
		PO x EC	< .01	.00	0.474	0.29
	**OP Allyship**	(Intercept)	5.29	0.06	< .001	
		Political Orientation	0.21	0.04	< .001	
		Economic Conservatism	-.01	.00	0.128	
		PO x EC	< .01	.00	0.979	0.16
	**OP Allyship - Transformed**	(Intercept)	29.03	0.6	< .001	

		Political Orientation	2.04	0.39	< .001	
		Economic Conservatism	-.06	0.04	0.087	
		PO x EC	-.01	0.01	0.677	0.15
**EA3 - Closeness**	**SP Allyship - Transformed**	(Intercept)	28.89	0.45	< .001	
		Political Orientation	3.17	0.26	< .001	
		Closeness	0.96	0.3	0.001	
		PO x Closeness	-.59	0.17	< .001	0.31
	**OP Allyship**	(Intercept)	5.30	0.05	< .001	
		Political Orientation	0.24	0.03	< .001	
		Closeness	0.24	0.03	< .001	
		PO x Closeness	-.04	0.02	0.031	0.28

Note. All continuous predictors were centered at their mean when interaction terms were included. SP = Self-perceived, OP = Other-perceived, PO = Political Orientation, SC = Social conservatism, EC = Economic conservatism. Age and conservatism are reflecting cisgender, straight participants' reports while closeness was measured among LGBTQ+ participants.

Components of allyship

We explored R2 across each individual component of allyship. See Table S5 for all model parameter estimates for each component model (non-prejudice, action, and humility).

Importantly, for non-prejudice and action models, there were statistically significant interactions between political orientation and other-perceived action or non-prejudice, such that the relationship between political orientation and self-perceived action or non-prejudice depended on the level of other-perceived action or non-prejudice.

For the non-prejudice model, simple slopes indicated that the relationship between political orientation was strongest when other-perceived non-prejudice was low (*b* = .24, *p* < .001), compared to average (*b* = .15, *p* < .001) and high (*b* = .07, *p* = .070). This pattern was also observed for the action model, such that the influence of political orientation was strongest when other-perceived action was low (*b* = .50, *p* < .001), compared to average (*b* = .41, *p* < .001) and high (*b* = .32, *p* < .001).

Non-prejudice and humility models violated the assumption that residuals are normally distributed (Shapiro-Wilk *W* = .95-.98, *ps* < .001), therefore, we employed a quadratic transformation of the outcome variables (i.e., other-perceived action and humility squared). Results from the transformed outcome variables were consistent with the untransformed results reported and are also included in Table S5.

Table S5

Components of allyship

**Outcome**	**Predictor**	***b***	***SE***	***p***	**Adj. R^2**
**SP Nonprejudice**	(Intercept)	6.18	0.04	< .001	
	Political Orientation	0.15	0.03	< .001	
	OP Nonprejudice	0.38	0.04	< .001	
	PO × SP Nonprejudice	-0.08	0.02	< .001	0.37
**SP Nonprejudice**	(Intercept)	39.04	0.48	< .001	
Transformed	Political Orientation	1.63	0.28	< .001	
	OP Nonprejudice	4.27	0.50	< .001	
	PO × SP Nonprejudice	-0.80	0.26	.002	0.36
**SP Action**	(Intercept)	4.67	0.07	< .001	
	Political Orientation	0.41	0.04	< .001	
	OP Action	0.51	0.04	< .001	
	PO × SP Action	-0.06	0.02	.011	0.55
**SP Humility**	(Intercept)	4.94	0.07	< .001	
	Political Orientation	0.09	0.04	.022	
	OP Humility	0.45	0.05	< .001	
	PO × SP Humility	-0.01	0.03	.629	0.18
**SP Humility**	(Intercept)	26.38	0.62	< .001	
Transformed	Political Orientation	0.66	0.36	.063	

OP Humility	4.26	0.49	< .001	
PO × SP Humility	0.04	0.28	0.881	0.18

Note. All continuous predictors were centered at their mean when interaction terms were included. SP = Self-perceived, OP = Other-perceived, PO = Political Orientation. All transformations performed were quadratic transformations of the outcome variable.

EA1 – Age

The model with other-perceived allyship as the outcome violated the normality of residuals. We performed a quadratic transformation of the outcome variable and report the results in Table S4.

EA2 – Social and economic conservatism

We wondered whether political orientation on a liberal-conservative continuum may be reductive and wanted to consider whether specific strains of conservatism (economic, social) may influence the relationship between political orientation and allyship differentially. Perhaps for conservatives, the relationship between their political orientation and allyship may be stronger for people who are more socially conservative and weaker for people who are more economically conservative. Allyship (self-perceived and other-perceived) was regressed on political orientation, the type of conservatism (social or economic), and their interaction. All predictors were mean-centered prior to analysis. Any statistically significant interactions were probed at high (+1 SD) and low (-1 SD) levels for social and economic conservatism.

Social Conservatism

Self-perceived allyship. The overall model for self-perceptions was statistically significant ($F(3, 371) = 51.20$, Adj. $R^2 = .29$, $p < .001$). Results revealed statistically significant main effects of political orientation and social conservatism of self-perceived allyship to the

LGBTQ+ community. Accounting for global political orientation, less socially conservative participants rated themselves better as allies. Results did not support an interaction between political orientation and social conservatism (p = .402).

Other-perceived allyship. The overall model for other-perceptions was statistically significant (F(3, 371) = 24.06, Adj. R^2 = .16, p < .001). Results supported only a significant main effect of political orientation. Social conservatism and the interaction between political orientation and social conservatism were not statistically significant predictors in this model.

This model violated the assumption that residuals are normally distributed (Shapiro-Wilk W = .98, p < .001), therefore, we employed a quadratic transformation of the outcome variable (i.e., other-perceived allyship squared). Results from the transformed outcome variable were consistent with the untransformed results reported are also included in Table S4.

Economic Conservatism

Economic conservatism had a poor alpha (α = .71) that fell below our pre-registered threshold (α = .80). No individual items appeared to be driving this poor internal consistency of the measure. Because we pre-registered these analyses, we ran them regardless and report them below.

Self-perceived allyship. The overall model was statistically significant (F(3, 371) = 52.82, Adj. R^2 = .29, p < .001). Results revealed statistically significant main effects of political orientation and economic conservatism of self-perceived allyship to the LGBTQ+ community. After controlling for political orientation, less economically conservative individuals tended to rate themselves as better allies to the LGBTQ+ community. Results did not support an interaction between political orientation and economic conservatism.

Other-perceived allyship. The overall model was statistically significant ($F(3, 371)$ = 23.94, Adj. R^2 = .16, p < .001). Results only supported a statistically significant main effect of political orientation predicting other-perceived allyship. Economic conservatism and the interaction between political orientation and economic conservatism were not supported as statistically significant predictors of other-perceived allyship to the LGBTQ+ community.

This model violated the assumption that residuals are normally distributed (Shapiro-Wilk W = .98, p < .001). We employed a quadratic transformation of the outcome variable (i.e., other-perceived allyship squared) and results were consistent with the untransformed results reported and are included in Table S4.

EA3 – Closeness

Self-perceived allyship

Residuals were not normally distributed in this model (Shapiro-Wilk W = .99, p = .006). We employed a quadratic transformation of the outcome variable (i.e., self-perceived allyship squared) and results were consistent with the untransformed results reported and are included in Table S4.

Other-perceived allyship

Residuals were not normally distributed in this model (Shapiro-Wilk W = .99, p = .002). We employed a quadratic transformation of the outcome variable (i.e., other-perceived allyship squared). Notably, results deviated from the untransformed results, such that the interaction term was no longer statistically significant (p = .081). Given this deviation, we align with the transformed results interpretation, which are reported in full in the manuscript; the unadjusted results are reported in full in Table S4.

EA4 - Authenticity

We pre-registered analyses using the Truth and Bias (T&B) model. However, our adaptation deviated from the traditional implementation, so we included additional measures (authenticity) to enable exploratory analyses using the standard T&B framework. Specifically, we examined self-perceived and other-perceived authenticity using the T&B model, regressing judgments (other-perceived authenticity) on truth (targets' self-perceived authenticity) and bias (evaluators' self-perceived authenticity). All variables were centered using the mean of the truth variable prior to analysis.

Model Specifications

- **Model 1:**

  Judgment of LGBTQ+ authenticity = Directional Bias + t(LGBTQ+ authenticity) + b(Cishet authenticity) + Error

- **Model 2:**

  Judgment of Cishet authenticity = Directional Bias + t(Cishet authenticity) + b(LGBTQ+ authenticity) + Error

Judgement of LGBTQ+ authenticity

The overall model was statistically significant, $F(2, 375) = 290.30$, $Adj.\ R^2 = .61$, $p < .001$. The estimate of directional bias (intercept) was −0.05, reflecting the difference between LGBTQ+ individuals' self-perceived authenticity and how authentic they were perceived to be by their cisgender, straight close other. This directional bias was not statistically significant different from 0 ($p = .139$), suggesting no systematic over- or underestimation.

The truth force parameter was 0.22 ($p < .001$), indicating that LGBTQ+ individuals’ self-reported authenticity significantly predicted how authentic they were perceived to be. This positive association reflects correlational accuracy—perceivers’ judgments aligned with the target's actual (self-reported) authenticity.

The bias force parameter was 0.64 ($p < .001$), indicating that the cisgender, straight individuals’ own self-reported authenticity influenced how authentic they judged their LGBTQ+ close other to be. This positive bias force suggests an *assumed similarity*—cisgender, straight individuals with high authenticity tended to perceive their LGBTQ+ close other as highly authentic.

Judgement of LGBTQ+ authenticity = -.05 + t(.22) + b(.64) + .68

Judgement of Cishet authenticity

This model was also statistically significant, $F(2, 375) = 330.80$, $Adj.\ R^2 = .64$, $p < .001$. The estimate of directional bias was −0.07, indicating that LGBTQ+ participants, on average, rated their cisgender, straight close other as less authentic than the cisgender, straight individuals rated themselves. This bias was statistically significant ($p = .031$), suggesting systematic underestimation of authenticity by LGBTQ+ perceivers.

The truth force parameter was 0.28 ($p < .001$), showing that cisgender, straight individuals’ self-reported authenticity significantly predicted how authentic they were perceived to be by their LGBTQ+ close other. As in the previous model, this reflects correlational accuracy.

The bias force parameter was 0.61 (p < .001), again suggesting an assumed similarity effect—LGBTQ+ participants with high authenticity tended to perceive their cishet close other as highly authentic.

Judgement of Cishet authenticity = -.07 + t(.28) + b(.61) + .67

EA5 - LGBTQ+ identity centrality

We also collected data on self-perceived and other-perceived identity centrality (i.e., the degree to which LGBTQ+ individuals see being part of the community as central to their identity). Self-perceptions (from LGBTQ+ participants) and other-perceptions (from cisgender, straight participants) of LGBTQ+ identity centrality were inputted into the same regression model as we executed for our RQ2 (i.e., self-perceived allyship regressed on other-perceived allyship, political orientation, and their interaction). Results for the full model are included in Table S6.

The overall model was statistically significant ($F(3, 371) = 62.39$, $Adj.\ R^2 = .33$, $p < .001$). Both political orientation and self-perceived identity centrality were statistically significant predictors of other-perceived identity centrality. The interaction between political orientation and identity centrality was not statistically significant ($p = .885$).

The residuals in this model violated normality (Shapiro-Wilk $W = .98$, $p < .001$). We tried a square-root transformation of the outcome variable, bias, but the residuals were still not normally distributed. Instead, a robust data transformation was performed using M-estimators (Huber weights), which down weight extreme cases in a dataset (please see OSF for code used to conduct this robust data transformation). The parameter Phi (ϕ) was adjusted iteratively, based on results of multivariate normality (i.e., multivariate kurtosis). After multivariate

normality was satisfied (b2p = 14.15, kurtosis = -1.51, p = .130), the null hypothesis was retained at ϕ of .02. Results of this transformed version of the model are reported in S6 and are consistent with the unadjusted version.

EA6 – Internal and external motivations

We wanted to explore whether different motivations to respond without prejudice (i.e., internal vs. external) were driving discrepancies in allyship perceptions. Results suggested that self-perceptions of these motivations from cisgender, straight participants were relatively consistent with LGBTQ+ identifying close others' evaluations, specifically for internal motivations ($r(376)$ = .62, 95% CI [.56, .68], p < .001). This was not the case for external motivations ($r(376)$ = .35, 95% CI [.26, .44], p < .001). Because of this discrepancy, we looked at self-perceived motivations and other-perceived motivations as separate predictors of bias (difference between self-perceived allyship and other-perceived allyship).

Our allyship bias score was regressed on cisgender, straight individuals' internal and external motivations to respond without prejudice to the LGBTQ+ community. All regression results are reported in Table S6.

Self-perceived motivations

The overall model was statistically significant ($F(2, 375)$ = 6.21, Adj. R^2 = .03, p = .002). Internal motivations to respond without prejudice, but not external motivations to respond without prejudice (b = .12, p = .206), was a statistically significant predictor of bias (i.e., overestimation). This finding suggests that the more internally motivated to avoid bias towards LGBTQ+ people, the more likely that individual is to overestimate their allyship relative to how they are perceived by LGBTQ+ close others.

This model violated the assumption that residuals are normally distributed (Shapiro-Wilk W = .98, p < .001). We tried a square-root transformation of the outcome variable, bias, but the residuals were still not normally distributed. Instead, a robust data transformation was performed using M-estimators (Huber weights), which down weight extreme cases in a dataset. The parameter Phi (ϕ) was adjusted iteratively, based on results of multivariate normality (i.e., multivariate kurtosis). After multivariate normality was satisfied (b2p = 14.13, kurtosis = -1.55, p = .120), the null hypothesis was retained at ϕ of .05. Results from the robust version of the data corresponded to our unadjusted results and are reported in Table S6.

Other-perceived motivations

The overall model was statistically significant (F(2, 375) = 11.52, Adj. R^2 = .05, p < .001). Perceived internal motivations to respond without prejudice (b = -.18, p < .001), but not external motivations to respond without prejudice (p = .687), was a statistically significant predictor of bias (i.e., overestimation). This finding contrasts with the finding above and suggests that the less internally motivated to respond without prejudice towards LGBTQ+ people someone is perceived to be, the more likely that individual is to overestimate their allyship relative to how they are perceived by LGBTQ+ close others.

This model also violated the assumption that residuals are normally distributed (Shapiro-Wilk W = .98, p < .001). A robust data transformation was performed using M-estimators (Huber weights). After multivariate normality was satisfied (b2p = 14.11, kurtosis = -

1.58, p = 0.110), the null hypothesis was retained at ϕ of .04. Results from the robust version of the data corresponded to our unadjusted results and are reported in Table S6[1].

EA7 – Interpersonal trust

Both models reported in the manuscript violated normality of residuals. We performed a quadratic transformation of the outcome variable and report the results in Table S6. Results are consistent with the untransformed versions reported in the manuscript.

EA8 – Allyship norm

We examined whether the discrepancy between how cisgender, straight individuals' allyship was perceived relative to a typical cisgender, straight individual in the LGBTQ+ identifying individuals' social network influenced perceptions of interpersonal trust for LGBTQ+ individuals. First, we calculated a difference score between other-perceived allyship and typically experienced cisgender, straight allyship, with higher scores reflecting greater perceived allyship from the cisgender, straight close other (M = .65, SD = 1.37, *range* = -3.64 – 5.18).

The overall model was statistically significant ($F(1, 376) = 24.85$, Adj. $R^2 = .06$, $p < .001$). Results supported a positive influence of perceived allyship of the cisgender, straight close other (vs. a typical cisgender, straight person) predicting interpersonal trust, such that greater interpersonal trust experienced by LGBTQ+ individuals was predicted by better evaluations of

[1] We wondered whether these different results could be explained by any statistically significant interactions between internal and external motivations (both self- and other-perceived) predicting bias. Interaction terms were not statistically significant in either model (ps ≥ .684).

allyship from their cisgender, straight close other relative to typical cisgender, straight individuals in their social network.

Residuals were not normally distributed in this model (Shapiro-Wilk $W = .98$, $p < .001$). We employed a quadratic transformation of the outcome variable (i.e., interpersonal trust squared). Results were consistent with the untransformed results and are reported in full in S6.

We also looked at these relationships using the interaction approach we used for EA7, where interpersonal trust was regressed on other-perceived allyship, allyship norm, and their interaction. The overall model was statistically significant ($F(3, 374) = 25.95$, Adj. $R^2 = .17$, $p < .001$). Only other-perceived allyship was a statistically significant predictor of interpersonal trust. Norms for cisgender, straight allyship for LGBTQ+ participants and their interaction were not statistically significant (*p*s > .05). This finding would suggest, as in our findings for EA7, how cisgender, straight individuals' allyship is perceived may be the driving factor of interpersonal trust.

Residuals were not normally distributed in this model (Shapiro-Wilk $W = .99$, $p = .002$). We employed a quadratic transformation of the outcome variable (i.e., interpersonal trust squared). Results were consistent with the untransformed results and are reported in full in Table S6.

Table S6

Regression results for EA5 - EA8

Exploratory Analysis	Outcome	Predictor	*b*	*SE*	*p*	Adj. R^2
**EA5**	**OP Identity Centrality**	(Intercept)	4.88	0.06	< .001	
		Political Orientation	0.10	0.04	0.009	
		SP Identity Centrality	0.53	0.04	< .001	
		PO × SP IC	< .01	0.02	0.885	0.33

	**OP Identity Centrality - Transformed**	(Intercept)	4.89	0.06	< .001	
		Political Orientation	0.08	0.04	0.021	
		SP Identity Centrality	0.53	0.04	< .001	
		PO × SP IC	< -.01	0.02	0.977	0.34
**EA6**	**Bias**	(Intercept)	-0.91	0.26	0.001	
		SP IMS	0.12	0.04	0.001	
		SP EMS	0.05	0.04	0.206	0.03
	**Bias - Transformed**	(Intercept)	-0.03	0.05	0.451	
		SP IMS	0.10	0.04	0.006	
		SP EMS	0.06	0.04	0.089	0.02
	**Bias**	(Intercept)	0.92	0.27	0.001	
		OP IMS	-0.18	0.04	< .001	
		OP EMS	0.02	0.04	0.687	0.05
	**Bias - Transformed**	(Intercept)	0.84	0.27	0.002	
		OP IMS	-0.17	0.04	< .001	
		OP EMS	0.02	0.04	0.686	0.05
**EA7**	**Interpersonal Trust - Transformed**	(Intercept)	31.92	0.27	< .001	
		Bias score	-2.24	0.51	< .001	0.05
	**Interpersonal Trust - Transformed**	(Intercept)	31.70	0.50	< .001	
		OP Allyship	3.91	0.52	< .001	
		SP Allyship	-0.32	0.54	0.551	
		SP Allyship x OP Allyship	0.39	0.32	0.234	0.17
**EA8**	**Interpersonal Trust**	(Intercept)	5.49	0.05	< .001	
		Bias score*	0.16	0.03	< .001	0.06
	**Interpersonal Trust - Transformed**	(Intercept)	30.85	0.52	< .001	
		Bias score*	1.74	0.34	< .001	0.06
	**Interpersonal Trust**	(Intercept)	5.58	0.04	< .001	
		OP Allyship	0.35	0.04	< .001	
		Allyship norm	-.05	0.03	0.186	
		OP Allyship x Allyship norm	0.02	0.03	0.488	0.17
	**Interpersonal Trust - Transformed**	(Intercept)	31.84	0.46	< .001	
		OP Allyship	3.91	0.45	< .001	
		Allyship norm	-.53	0.38	0.158	
		OP Allyship x Allyship norm	0.30	0.30	0.316	0.17

Note. All models including interactions had predictors that were mean-centered prior to analysis. SP = Self-perceived, OP = Other-perceived, PO = Political Orientation (Cishet), IMS = Internal motivations to respond without prejudice, EMS = External motivations to respond without prejudice, Allyship norm = Allyship rating for a typical cisgender, straight person in LGBTQ+ social network.

*bias here is the difference score between other-perceived allyship and perceptions of a typical cisgender, straight person in the LGBTQ+ close others' network.
